# Sensitivity of Al-Doped Zinc-Oxide Extended Gate Field Effect Transistors to Low-Dose X-ray Radiation

**DOI:** 10.3390/ma16051868

**Published:** 2023-02-24

**Authors:** Amal Mohamed Ahmed Ali, Naser M. Ahmed, Norlaili A. Kabir, Ahmad M. AL-Diabat, Natheer A. Algadri, Ahmed Alsadig, Osamah A. Aldaghri, Khalid H. Ibnaouf

**Affiliations:** 1 School of Physics, Universiti Sains Malaysia, Gelugor 11800, Penang, Malaysia; 2 Department of Medical Instrumentation Engineering, Dijlah University College, Baghdad 11622, Iraq; 3 Department of Physics, Al-Zaytoonah University of Jordan, Amman 11733, Jordan; 4 Department of Physics, Isra University, Amman 00964, Jordan; 5 CNR NANOTEC Institute of Nanotechnology, Via Monteroni, 73100 Lecce, Italy; 6 Physics Department, College of Science, Imam Mohammad Ibn Saud Islamic University (IMSIU), Riyadh 13318, Saudi Arabia

**Keywords:** AZO, chemical bath deposition, radiation detector, EGFET, MOSFET

## Abstract

Herein, we investigated the applicability of thick film and bulk disk forms of aluminum-doped zinc oxide (AZO) for low-dose X-ray radiation dosimetry using the extended gate field effect transistor (EGFET) configuration. The samples were fabricated using the chemical bath deposition (CBD) technique. A thick film of AZO was deposited on a glass substrate, while the bulk disk form was prepared by pressing the collected powders. The prepared samples were characterized via X-ray diffraction (XRD) and field emission scanning electron microscope (FESEM) to determine the crystallinity and surface morphology. The analyses show that the samples are crystalline and comprise nanosheets of varying sizes. The EGFET devices were exposed to different X-ray radiation doses, then characterized by measuring the I–V characteristics pre- and post-irradiation. The measurements revealed an increase in the values of drain–source currents with radiation doses. To study the detection efficiency of the device, various bias voltages were also tested for the linear and saturation regimes. Performance parameters of the devices, such as sensitivity to X-radiation exposure and different gate bias voltage, were found to depend highly on the device geometry. The bulk disk type appears to be more radiation-sensitive than the AZO thick film. Furthermore, boosting the bias voltage increased the sensitivity of both devices.

## 1. Introduction

Zinc oxide (ZnO) in various forms has been used in a wide range of applications due to its low cost and biocompatibility [1]. The electrical and optical properties of ZnO influence the deposition techniques (e.g., chemical bath deposition (CBD), chemical vapor deposition (CVD) and radio frequency sputtering technique (RF)) as well as the doping process. Particularly, ZnO is employed as a sensitive material in the fabrication of gas, humidity and temperature sensors [2]. Aluminum-doped ZnO (AZO) films have received a great deal of interest as they are used as conductive electrodes due to their high conductivity and strong optical transmittance. Also, since it is less expensive than conventional crystals used for gamma and X-ray dosimetry research, and exists as high-surface-area nanostructures, it is ideal for dosimetric application [3]. Al is a typical dopant that functions as a donor in a ZnO lattice to encourage broad bandgap engineering (i.e., produce defects), which enhances the electrical and optical properties of ZnO [4]. Furthermore, Al-doped zinc oxide is extremely useful in the fabrication of optoelectronic devices such as pn-junction and superlattices, as well as detectors [5,6]. Exposure to ionizing radiation can occur in a variety of sectors, including healthcare, research and educational organizations and nuclear fuel operations. Hence, dosimetry is essential for the safe and appropriate use of radiation, radioactive materials and nuclear energy. The type of detector used for an application is determined by the required energy range (X- or γ-ray), resolution and efficiency parameters. Other factors to consider are detector efficiency, reaction time and environmental compatibility [7]. Due to their fascinating merits, metal oxide semiconductor field effect transistors (MOSFETs) are a popular choice among commercially available dosimeters. Advantages include direct and non-destructive readings of dosimetry data, low energy consumption, straightforward calibration, appropriate sensitivity and reproducibility, small sensor dimensions, significantly wider dose range, compatibility with microprocessors and affordable price [8,9].

Several radiation-sensitive metal-oxide field effect transistor (RADFET) dosimetry sensors have been developed in recent decades, in line with rapid improvements in related technology [10]. However, RADFETs cannot capture individual X-ray photon incidents, are non-reusable and have no inherent energy resolution [11]. For these reasons, RADFETs were replaced with external gate field effect transistors (EGFETs) to connect the gate (the prepared device) externally. In an EGFET device, the detecting element is directly subjected to X-ray irradiation, while the MOSFET is totally isolated. In addition to the conventional MOSFET configuration, the EGFET structure offers a number of advantages, such as ease of handling, compactness, quickness in dosage readout, accuracy and low sensitivity to fluctuations in external circumstances during irradiation measurements [12]. Furthermore, the oxide gate layer of an EGFET may be fabricated using various methods, such as chemical bath deposition, implying that it can be produced using different metal oxide materials with varying thicknesses for in vivo dosimetry. More appealing, an improved public and occupational safety in high-radiation locations is possible with the use of wireless, real-time radiation EFGET sensor networks.

It is thought that ionizing radiation causes structural defects (referred to as color centers or oxygen vacancies in oxides) that induce a change in density. Radiation alters the quantity of charge carriers in semiconductor materials, thereby changing their properties. This change offers information on the dosage absorbed by the exposed materials. The quantifiable absorbed dose depends on the kind of radiation, its mode and rate of interaction with the components, the material characteristics, their specific contribution to the device performance and the physical principles underlying the function of the device [13,14].

ΔV_TH_ is calculated by measuring the effect on drain current versus gate voltage (I_d_–V_g_) characteristics of such a transistor. Nevertheless, ΔV_TH_ extraction with a device operating in output characteristic is also common. The MOSFET reading equipment was used to monitor the threshold voltage before and during exposure to radiation, which is proportional to the cumulative dosage,

∆V_TH_ = V_T_ − V_T0_ (1)

where ∆V_TH_ is the threshold voltage after the irradiation and V_T0_ denotes the threshold voltage before the irradiation. For this method, the system is biased to work in the transfer characterization by applying a bias voltage of 3, 5 and 7 V. The radiation sensor EGFET works by translating the radiation-induced threshold voltage shift, V_TH_, into the absorbed radiation dose *D*, which is expressed as

(2) $$\Delta V_{\mathrm{TH}}=S\times D^{n}$$

where *S* is a constant and *n* represents the degree of linearity, which depends on the electrical field, thickness of the oxide layers and absorbed radiation dose [15]. If *n* = 1, then *S* represents the sensitivity [16],

(3) $$\left(S\;=\;\Delta V_{TH}\cdot D^{-1}\right).$$

The radiation response of EGFET is a consequence of a complex interaction of energy-dependent processes: (1) electron–hole production, (2) electron–hole recombination, (3) hole transportation, (4) severe hole trapping as well as (5) radiation-induced interface traps and positive oxide trapped charge. These processes cause a shift in the threshold voltage, $\Delta V_{\mathrm{TH}}$ [17].

**Figure 1:**
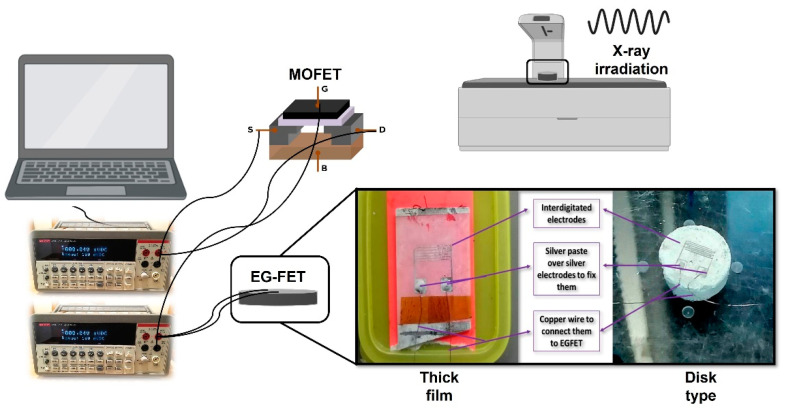
Schematics illustrating the EGFET connection setup and the key components used for this work: a commercial MOSFET (CD4007UB) is connected to the sensing component (the fabricated semiconductor gates) which interacts directly with X-ray radiation. The sensing layer was exposed to X-rays before being attached to the measuring equipment, then examined using a Keithley 2400 and Lab Tracer 2 software.

Up to now, limited research has been conducted on ZnO doped with other metals as semiconductor radiation detectors. Despite the commercial availability and functionality of the scintillation detectors (inorganic scintillators), the huge size and the requirement of extra cooling to function at high temperatures necessitate the investigation of efficient alternatives. To this end, most previous studies of MOSFETs as radiation monitors used silicon dioxide (SiO_2_) [18]. However, the use of ZnO-Al, in particular, as semiconductor detectors or EGFETs for X-ray detection has not yet been explored. Herein, we aim to investigate the sensitivity of the thick film and bulk disk forms of ZnO doped with Al (AZO) connected to the FET device to monitor the radiation dosage generated from kilovoltages of X-rays from a diagnostic X-ray machine (Figure 1). In addition, the effect of different bias voltages under different radiation doses on the sensitivity of prepared AZO (of various thicknesses) was studied.

## 2. Material and Methods

### 2.1. Deposition of ZnO Seed Layer on a Glass Substrate

The seed layer was prepared using the radio-frequency sputtering technique (RF). A 200 nm seed layer of ZnO was deposited by RF reactive sputtering (CESAR RF Power Generator) on glass substrates and gave an off-white color. The sputtering process was carried out at a high power of 150 W, pressure of ~5 × 10^−5^ mbar, and sputtering rate of 0.8 Å·s^−1^. Moreover, Ar gas flow was adjusted to 14 sccm, and the sputtering process was carried out at room temperature. The RF sputtering machine used was located at Nor Lab in the School of Physics, Universiti Sains Malaysia (USM), Penang, Malaysia. 

### 2.2. Synthesis of ZnO-Doped Al

**Figure 2:**
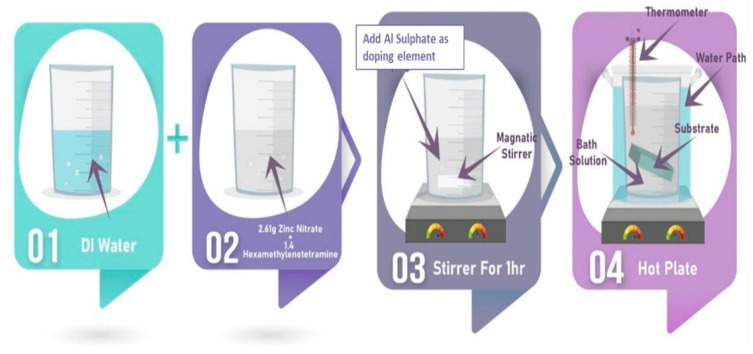
The steps followed for the preparation of AZO via CBD approach.

**Figure 3:**
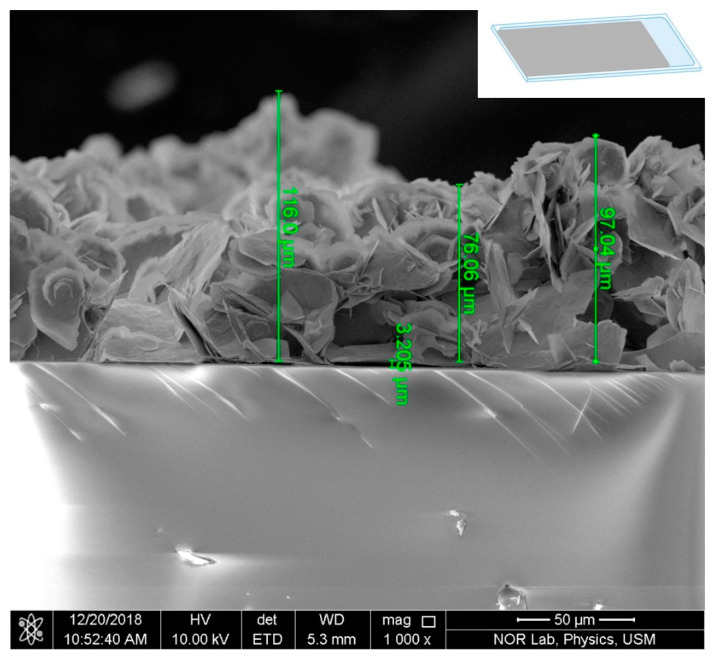
SEM micrograph displaying the cross section of the AZO thick film.

The chemical bath deposition (CBD) method was used to synthesize ZnO-Al thick film and disk powder types. Firstly, 2.62 g of zinc nitrate hexahydrate ($\mathrm{Zn}\;{\left({\mathrm{NO}}_{3}\right)}_{2}\cdot 6H_{2}O$) ($\mathrm{MW}=261.44\;g\cdot {\mathrm{mol}}^{-1}$) and 1.4 g of hexamethylenetetramine ($C_{6}H_{12}N_{4}$) $(\mathrm{MW}=140.19\;g\cdot {\mathrm{mol}}^{-1}$) were dissolved in deionized water (DIW) in a final volume of 200 mL, and left to react under stirring for 1 h. Next, 3.42 g of aluminum sulfate (${\mathrm{Al}}_{2}{\left({\mathrm{SO}}_{4}\right)}_{3}$) $(\mathrm{MW}=342.15\;g\cdot {\mathrm{mol}}^{-1}$) was added to the solution and kept under stirring for another 1 h. Following that, the pre-prepared 200 nm thin ZnO seed layer deposited on a glass substrate via RF sputtering was immersed in the solution with the coated surface facing the bottom of the beaker. The temperature of the solution was maintained at 90 °C for 7 h, as shown in Figure 2. The thick AZO film substrate was then washed with DIW and air-dried at room temperature. To enhance the thickness of the film, the preceding steps were repeated three times to make three layers. As determined by scanning electron microscopy (SEM), the combined thickness of the three layers was approximately 96.36 µm. (Figure 3). The mixture was then filtered at ambient temperature and dried in an oven at 80 °C for 20 min. After that, the powder was pressed to a thickness of 1 mm using a high-pressure hydraulic press. RF sputtering was used to deposit the fingerprint interdigitated silver electrodes on the thick film and disk samples, and wires were connected using the silver paste (see Figure 1).

### 2.3. Setup and Connection

The commercial MOSFET (CD4007UB) is connected to the sensing component (fabricated semiconductor gates) through a wire to keep the transistor away from irradiation. The sensing component interacts directly with X-ray radiation, as seen in Figure 1. The applied bias voltage used in this work is drain-to-source voltage (0.3, 1 and 2 V) when the system is connected to the linear regime. On the other hand, when the system is connected to the saturation regime, the applied bias voltage is gate-to-source voltage (3, 5 and 7 V). The sensing layer is exposed to X-rays before being attached to the measuring equipment, then examined under irradiation using a Keithley 2400 and Lab Tracer 2 software.

### 2.4. Characterization of the Prepared AZO Structures

The morphology and crystal structures of the prepared thick film and disk type AZO were investigated using field emission scanning electron microscopy (FESEM, FEI Nova NanoSEM, FEI Company, CA, USA) and X-ray diffraction (XRD, X’Pert PRO, PANalytical, Malvern PANalytical, UK) at a 2θ range of 20–80°. The I–V characteristics of the AZO thick film and the disk form sensors were recorded under irradiation exposure using two units of Keithley 2400 Source Measure Units (Keithley Instruments Inc., Cleveland, OH, USA), both connected to a computer and the drain–source input of the commercial CD4007 MOSFET device (Texas Instruments, Dallas, TX, USA). The prepared AZO samples were connected as an extended gate to acquire input from the MOSFET. For the setup used in this work, the voltage was operated in the range of 0–5 V at gate bias voltages of 3, 5 and 7 V for the saturation regime and 0.3, 1 and 3 V for the linear regime. From the linear regime, the relation between voltage and dose was plotted. The threshold voltage shift was measured under different bias voltages and radiation doses. On the other hand, the relationship between radiation dose under different gate bias voltages and the current were plotted from the saturation regime. In addition, the prepared samples were irradiated with varying doses of X-ray (9, 36.5 and 70 mGy). Further details on the experimental part are provided in a previous study [12]. Finally, the sensitivity of the AZO thick film and disk type dosimeters to different bias voltages under irradiation was studied and compared to find the most suitable dosimeter for low X-ray dose detection. All measurements were performed at room temperature.

## 3. Results and Discussion

### 3.1. Structural Profile of AZO Thick Film and Disk Type Samples

**Figure 4:**
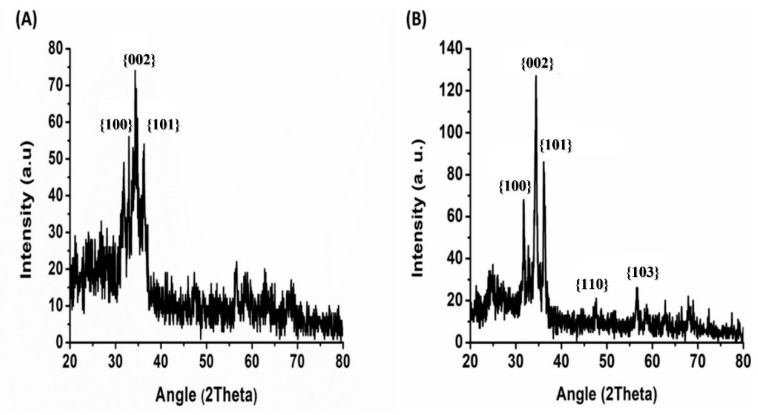
XRD patterns of AZO for (**A**) thick film and (**B**) disk type samples used in this work.

**Figure 5:**
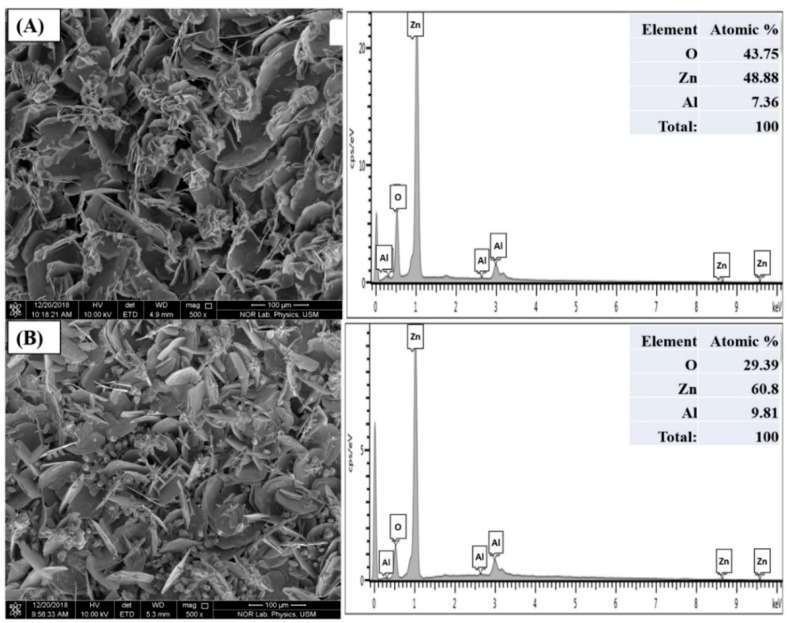
FESEM images with EDX of AZO for (**A**) thick film and (**B**) disk type samples.

XRD shows the crystalline phases of the AZO thick film and AZO disk type samples, whereas FESEM with EDX reveals their surface morphology and elemental composition (atomic percentages). Figure 4A,B illustrate the XRD patterns of the prepared AZO thick film and disk type sensors, respectively. The XRD results demonstrate that AZO thick films have a crystalline structure and display ZnO peaks with lower intensities than AZO disk. The peaks occur at five values of 31.7, 34.5, 36.2, 51.4 and 56.9°, which correspond to {100}, {002}, {101}, {110} and {103}, respectively. The FESEM micrographs of AZO thick film display nanosheets of different diameters, as shown in Figure 5.

### 3.2. Impact of X-ray Irradiation and the Bias Voltage on AZO Dosimeter

**Figure 6:**
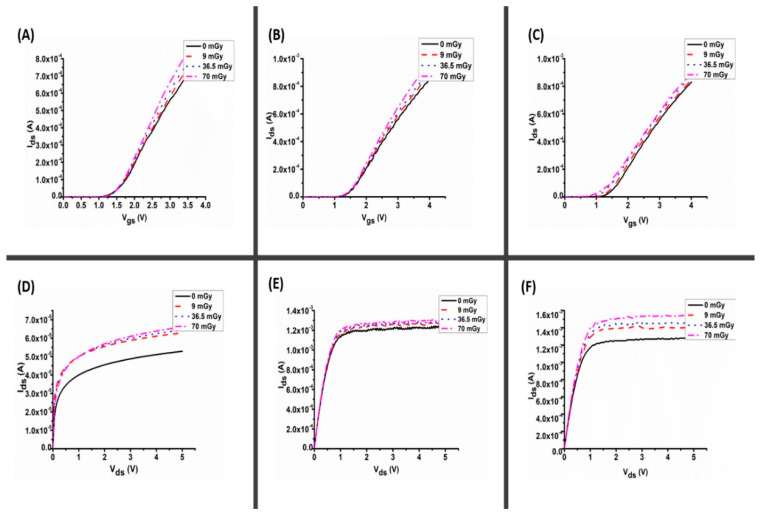
I–V characterization of AZO thick film for (**A**–**C**) transfer characteristics for bias voltage 0.3, 1 and 3 V, respectively, and (**D**–**F**) output characteristics for gate bias voltage 3, 5 and 7 V, respectively.

**Figure 7:**
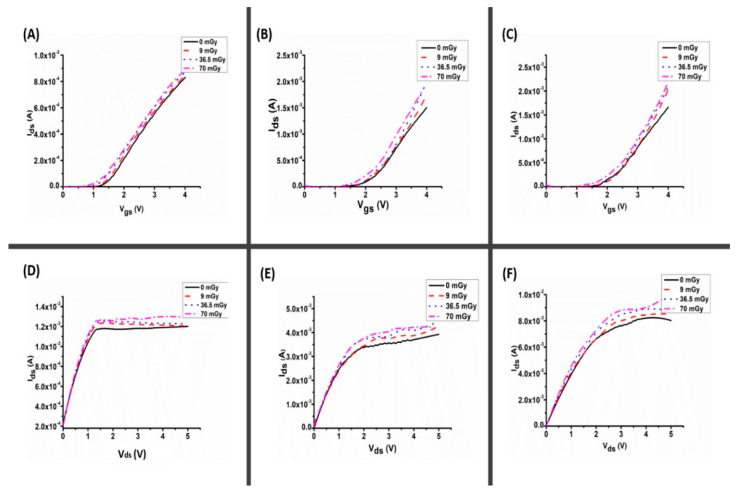
I–V characterization of AZO (disk type) for (**A**–**C**) transfer characteristics for bias voltage 0.3, 1 and 3 V, respectively, and (**D**–**F**) output characteristics for bias voltage 3, 5 and 7 V, respectively.

Figure 6 and Figure 7 show the current–voltage (I–V) curves for the fabricated thick film (96.36 µm) and disk type (1.0 mm) AZO samples, respectively, before and after X-ray irradiation at various doses (9, 36.5 and 70 mGy). Figure 6 shows radiation-induced changes on AZO thick film and its current under different applied bias voltages of 0.3, 1 and 3 V for the transfer characteristics (Figure 6A–C), and 3, 5 and 7 V for output characteristics (Figure 6D–F). Similarly, Figure 7 depicts the same set of parameters for disk-type samples. The results reveal that exposing the samples to low-dose X-rays significantly improves their current–voltage characteristics (see also transfer characterizations Figure S1). Under bias voltages of 3, 5 and 7 V, the changes in current (∆I) for the thick film are 11.3 µA, 70 µA and 270 µA, all within ± 0.85 µA. Under the same bias voltages, the ∆I for the disk type (1 mm) AZO was found to be 110 µA, 530 µA and 890 µA, within ± 1.38 µA (3, 5 and 7 V). The current magnitude under the biased condition increased significantly as the radiation dosage increased to 70 mGy. The generation lifetime, which determines the rate of hole emission in the depletion area, is the parameter that governs the current [13]. This considerable change in the current for thick films and 1 mm thickness (disk form) indicates that this thickness (i.e., 1 mm) is suitable for manufacturing a radiation detector under this low dosage of X-ray radiation. The increase in the drain current may be attributed to the large number of oxide charges induced by irradiation, which increased the conductivity of the device. The increase in conductivity led to a decrease in drain-to-source resistance and an increase in the drain current [19]. Therefore, the impact of X-ray radiation on the films is dependent on both the dose and characteristics of the films, particularly their thickness. The degradation is more severe for greater dose and thicker films [20].

**Figure 8:**
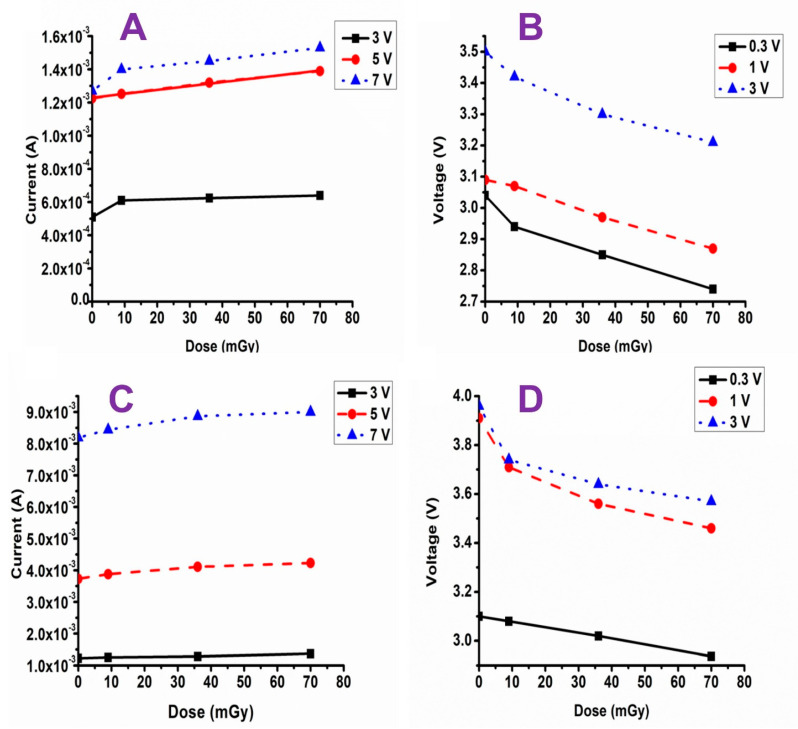
The effect of bias voltage on (**A**,**C**) current vs. dose and (**B**,**D**) voltage vs. dose for AZO thick film and AZO disk type, respectively.

Figure 8A,C illustrate the increase in current values with increasing doses of radiation up to 70 mGy at bias voltages of 3, 5 and 7 V. The filling of vacant states may account for the observed increase in current values with increased radiation exposure. [21]. On the other hand, Figure 8B,D show the output of the device (voltage shift) as a factor of total absorbed dose for 0.3, 1 and 3 V bias devices. The data also show that increasing the radiation dose led to degradation of the output voltage. In contrast, increasing the thickness of the film concomitantly increased the output voltage for all applied bias voltages.

**Figure 9:**
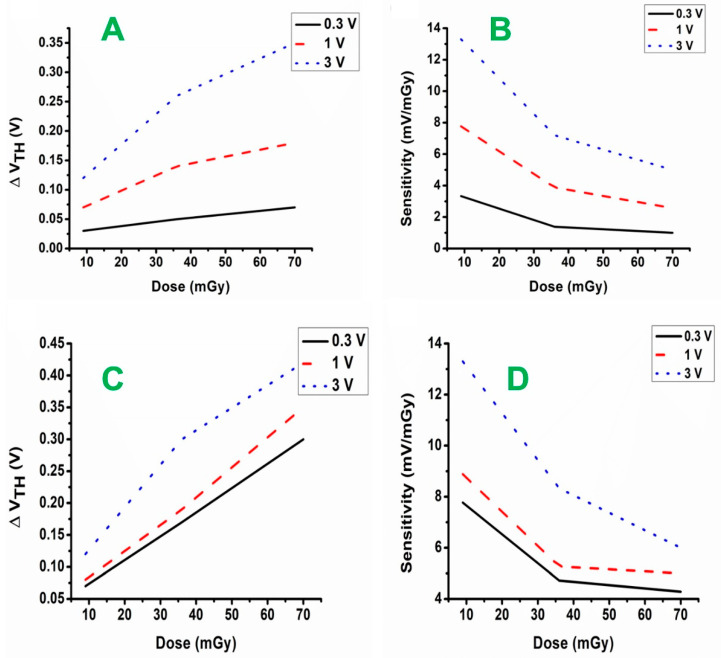
The effect of bias voltage on (**A**,**C**) threshold voltage with dose and (**B**,**D**) sensitivity with dose for thick film and disk type AZO, respectively.

Figure 9A,C show the impact of applied bias voltages under different X-ray doses on the threshold voltage of the samples. The figures also extrapolate ∆V_TH_ during irradiation for thick film and disk type samples. In all cases, the similarity between extrapolated ∆V_TH_ for different bias voltages is excellent, supporting the EGFET design’s usage in real applications. For the same dose interval, increasing the gate bias from 0.3 V to 3 V increased the ∆V_TH_ by approximately 9%; ∆V_TH_ of 0.3 V was 0.03 V, while the ∆V_TH_ of 3 V was 0.12 V for a dose of 9 mGy. The results also show that increasing the thickness of AZO gate oxide layer significantly increased the ∆V_TH_ for the same radiation dosage, which is largely due to an increase in fixed trap density [22]. Through a screening mechanism, the upsurge in oxide and interface charges affects the effective bias electric field across the sensitive layer, affecting electron–hole recombination and charge yield. Fewer holes are trapped at the surface due to electron–hole recombination.

Figure 9B,D show that the dose sensitivity of EGFET decreased with an increase in radiation, although this occurred at the same rate for all gate bias voltages, the results were tabulated in Table 1. As observed, the variation in threshold voltage became more evident as the radiation dose increased. As predicted, providing a positive voltage to the gate increased the concentration of positive oxide-trapped charges, enhancing the threshold voltage shift of EGFETs. This is because a greater electric field in the oxide reduces the chances of electron–hole recombination due to bond breakage in the oxide. Electrons exit the oxide easily in the active phase and thus can be collected by the gate [23]. One feature of EGFET dosimetry observed with each particular dosimeter was an intrinsic reduction in sensitivity with applied dosage. This is attributable to changes in the effective electric field supplied to the EGFET during irradiation, which results in a buildup of holes at the AZO interface [24]. The threshold voltage shifted for the same rise in dosage as gate bias increased; therefore, the dosimeter’s sensitivity increased as the gate bias voltage continued to rise. However, the sensitivity was also decreased with an increase in radiation dose. The linear response of threshold voltage shift is essential to prove the dosimeter’s linearity performance for different irradiation levels. Even with MOSFETs explicitly developed for commercial radiation dosimeters, achieving better linearity is difficult. Because the Z_eff_ of AZO (Z_eff_ = 25.39) is higher than that of SiO2 (Z_eff_ = 18.99) used in the MOSFET, the EGFET interacts with radiation through a photoelectric effect and produces more electrons, which leads to an increase in the current [25]. The higher Z_eff_ value of AZO is crucial, since photoelectric interaction is greatly influenced by the atomic number of a material. There is an excellent response of EGFETs at low energies (100 keV) because of the dominance of the photoelectric effect in this range of energies [26,27]. In addition, due to the larger photoelectric cross section of metals than metal oxides, secondary electrons generated in the metal will pass into metal oxide and contribute to the absorbed dose.

## 4. Conclusions

This study investigated the possibility of using AZO thick films and disk-type AZO as sensitive materials to measure low X-ray dosage. Interdigitated planar structured devices based on AZO were fabricated via CBD. All devices were subjected to radiation dosages of 9, 36 and 70 mGy. The electrical features of the films were strongly dependent on the radiation doses and bias voltage. Thus, the feasibility of building an EGFET-based device is dependent on the sensitivity to X-ray irradiation and operating dosage area. The current values of the AZO thick film (with interdigitated electrodes) increased with radiation exposure, as well as with the bias voltage from 3 V to 7 V. On the other hand, increasing the thickness of the device from a 98 μm thick film to a 1 mm disk led to an increase in the sensitivity of the device. Thus, sensitivity to X-radiation exposure is highly dependent on the design of the sensing device, while the bulk disk type showed a higher sensitivity to radiation than the AZO thick film. In contrast, increasing the radiation exposure dose reduced the efficiency and sensitivity of the device. The proposed implementation could be considered as potential choices for low-cost, real-time X-ray dosimetry.

## Figures and Tables

**Table 1:** The sensitivity of thick film and disk type dosimeters exposed to different radiation doses under an applied gate bias voltage (0.3, 1 and 3 V).

Radiation Dose (mGy)	Sensitivity of 98 μm Thick Film Dosimeter (mV/mGy)			Sensitivity of 1 mm Disk Type Dosimeter (mV/mGy)		
	0.3 V	1 V	3 V	0.3	1 V	3 V
9	3.33	7.77	13.3	7.77	8.88	13.3
36.5	1.38	3.88	7.2	4.72	5.27	8.33
70	1	2.57	5	4.28	5	6

## Data Availability

Not applicable.

## Supplementary Materials

The following supporting information can be downloaded at: https://www.mdpi.com/article/10.3390/ma16051868/s1, Figure S1: Transfer characteristics in the y-log scale for the used biased voltages, the graphs give more data concerning off-current, subthreshold swing, and threshold voltage.
