# Supplementary material for: Sensitivity of Al-Doped Zinc-Oxide Extended Gate Field Effect Transistors to Low-Dose X-ray Radiation
Source: Materials (Basel). 2023 Feb 24;16(5):1868. doi: 10.3390/ma16051868 (PMC10004138; doi:10.3390/ma16051868)
Supplement: Supplementary file 1 [file materials-16-01868-s001.zip › materials-2124716-supplementary.pdf]

# Sensitivity of Al-Doped Zinc-Oxide Extended Gate Field Effect Transistors to Low-Dose X ray Radiation

**Amal Mohamed Ahmed Ali[1], Naser M. Ahmed[1,2*], Norlaili A. Kabir [1], Natheer A. Algadri [3], Ahmad M. AL-Diabat [4], Ahmed Alsadig [5], Osamah A. Aldaghri[6] and Khalid H. Ibnaouf [6,*]**

[1,] School of Physics, Universiti Sains Malaysia 11800, Penang, Malaysia
[2,] Department of medical instrumentation engineering, Dijlah university college, Baghdad, Iraq
[3]Department of Physics, Isra University, Amman, Jordan.
[4]Department of Physics, Al-Zaytoonah University of Jordan, Amman, Jordan.
[5]CNR NANOTEC Institute of Nanotechnology, via Monteroni, 73100 Lecce, Italy
[6]Physics Department. College of Science, Imam Mohammed Ibn Saud Islamic University (IMSIU), Riyadh 13318, Saudi Arabia

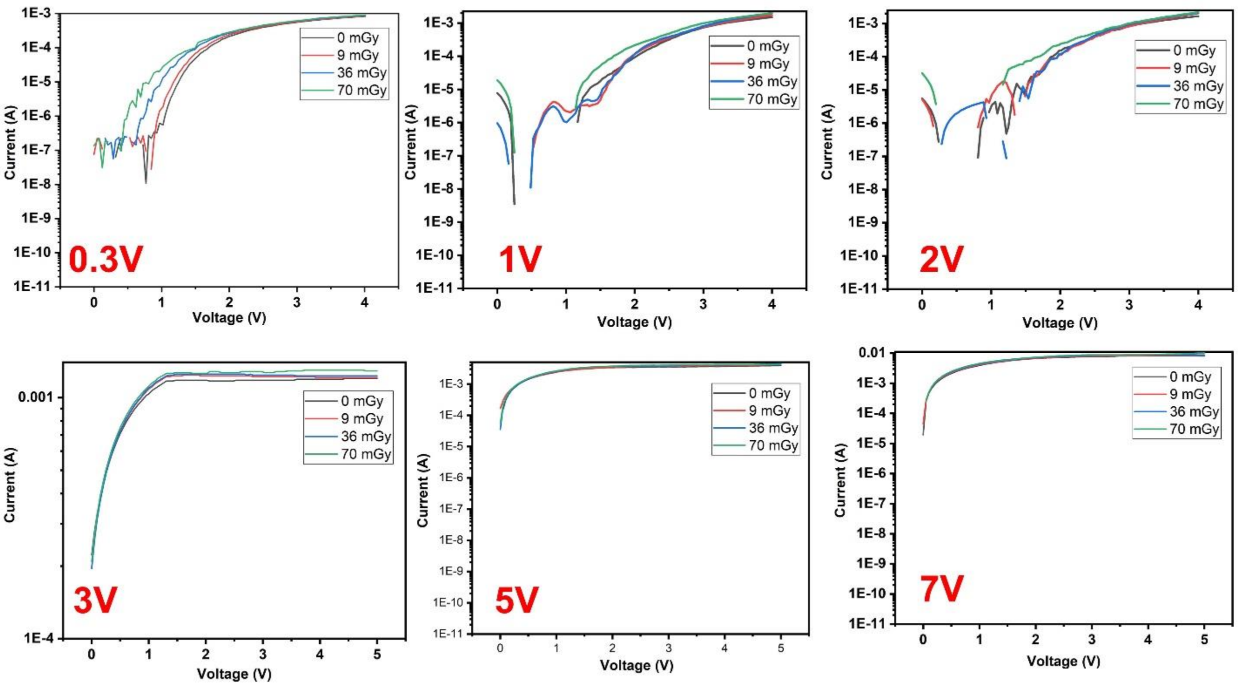


**Figure S1.** Transfer characteristics in the y-log scale for the used biased voltages, the graphs give more data concerning off-current, subthreshold swing, and threshold voltage).
